# Polypharmacy in the treatment of people diagnosed with borderline personality disorder: repeated cross-sectional study using New Zealand's national databases

**DOI:** 10.1192/bjo.2023.592

**Published:** 2023-10-26

**Authors:** Matthew Tennant, Chris Frampton, Roger Mulder, Ben Beaglehole

**Affiliations:** Department of Psychological Medicine, University of Otago, Christchurch, New Zealand

**Keywords:** Borderline personality disorder, polypharmacy, antidepressants, antipsychotics, comorbidity

## Abstract

**Background:**

There is insufficient evidence to support the pharmacological treatment of borderline personality disorder. However, previous out-patient cohorts have described high rates of polypharmacy in this group. So far, there have been no national studies that have considered polypharmacy in borderline personality disorder.

**Aims:**

To describe psychotropic polypharmacy in people with borderline personality disorder in New Zealand.

**Method:**

New Zealand's national databases have been used to link psychotropic medication dispensing data and diagnostic data for borderline personality disorder. Annual dispensing data for 2014 and 2019 have been compared.

**Results:**

Fifty percent of people with borderline personality disorder who were dispensed medications had three or more psychotropic medications in 2014. This increased to 55.9% in 2019 (*P* < 0.001). Those on seven or more psychotropics increased from 8.4 to 10.7% (*P* < 0.023). Quetiapine was the most dispensed psychotropic medication, being given to 53.8% of people dispensed medication with borderline personality disorder in 2019. Lorazepam dispensing showed the largest increase, going from 15.5 to 26.7% between 2014 and 2019 (*P* < 0.001).

**Conclusions:**

There is a large burden of psychotropic polypharmacy in people with borderline personality disorder. This is concerning because of the lack of evidence regarding the efficacy of these medications in this group.

The recommended treatments for borderline personality disorder are psychological and psychosocial interventions.^1^ There is insufficient evidence to support the pharmacological treatment of borderline personality disorder. No psychotropic drug has been licensed for borderline personality disorder, and medications do not change the natural course of the disease.^2^ The 2009 National Institute for Health and Care Excellence (NICE) guidelines stated that individuals with borderline personality disorder and no active psychiatric comorbidity should be encouraged to reduce or stop pharmacotherapy.^1^ Despite this lack of evidence, psychotropic medications are frequently used in the treatment of borderline personality disorder. The most common medications prescribed are antidepressants, anxiolytics, antipsychotics and mood stabilisers. Cohort studies of selected out-patient units have looked at polypharmacy in people with borderline personality disorder in Spain^3,4^ and the UK,^5^ and reported high rates of psychotropic polypharmacy. Cohorts from psychiatric hospitals in Europe and the USA^6–8^ had similar findings. So far, there have been no national cohort studies that have considered polypharmacy in borderline personality disorder, and to our knowledge, this is the first study to describe national dispensing trends for people with borderline personality disorder. The study evaluates change in psychotropic dispensing in New Zealand between 2014 and 2019.

## Method

Ethics approval was provided by the Otago University Ethics Committee (HD21/095) under the Minimal Risk Health Research Pathway. Data had been collected and stored within national databases. It was received and analysed in a deidentified form, using a unique identifier.

### National databases

The New Zealand Ministry of Health collects psychiatric diagnoses and demographic information for people by using public sector secondary care and non-governmental organisation mental health and addiction services within the Programme for the Integration of Mental Health Data (PRIMHD) database. National pharmaceutical dispensing data for New Zealand is recorded in the Pharmaceutical Collection database. This includes prescriptions from both public and private clinicians.

### Data linkage

PRIMHD data were requested for all patients that received a diagnosis of borderline personality disorder in a 10-year period, from 1 January 2009 until 31 December 2019. Unique identifiers linked diagnosis to demographic information, including age, gender, ethnicity and New Zealand index of socioeconomic deprivation (NZDep) score. The NZDep is an area-based measure of socioeconomic status. It is expressed in deciles, where 10 indicates the highest level of socioeconomic deprivation.

Dispensing data for the patients identified in the PRIMHD sample were requested with the same unique identifier, to allow the data-sets to be linked. For those with a diagnosis of borderline personality disorder between 2009 and 2014, annual dispensing of psychiatric medications was requested for 2014. For those with a diagnosis of borderline personality disorder between 2015 and 2019, annual dispensing of psychiatric medications was requested for 2019. Annual dispensing was defined as being dispensed any of the included medications at least twice within the calendar year.

### Medication classification

Psychotropic medications were classified according to the Pharmac Medication Schedule,^9^ with the exception of the ‘mood stabilisers’, which comprised selected anti-epileptic medications and lithium carbonate, based on common clinical use. Benzodiazepines and zopiclone were considered separately to other anxiolytic and hypnotic agents (e.g. melatonin) because of their distinctive pharmacological profiles and clinical importance. The psychotropic medications included in the study are outlined in Appendix 1.

### Data analysis

Psychotropic polypharmacy was defined as being dispensed three or more psychotropic medications. Those on seven or more psychotropic medications were considered to have extensive polypharmacy. We examined the percentage of people with borderline personality disorder who had polypharmacy and extensive polypharmacy in 2014 compared with 2019. The percentage of individuals who were dispensed each psychotropic medication was also reported. Chi-squared tests were used to compare the key polypharmacy percentages between the two cohorts.

## Results

Table 1 reports demographic details for the study populations between 2009 and 2014, and 2015 and 2019. The number of people with a diagnosis of borderline personality disorder between 2009 and 2014 was 1932, of whom 1468 were dispensed psychotropic medications. Therefore, 464 (24.0%) were dispensed no psychotropic medications in 2014. Between 2015 and 2019, the number of people with a diagnosis increased to 2368, of whom 1915 were dispensed psychotropic medications. Those dispensed no psychotropic medications reduced to 453 (19.1%) in 2019. There was a predominance of females in both samples: 82.6% in 2009–2014 and 85.4% in 2015–2019. The majority in both groups identified as New Zealand European. The mean age was 37.6 years in the 2009–2014 group and 33.6 years in the 2015–2019 group.

**Table 1:** Demographics of those dispensed psychotropic medications

	2009–2014		2015–2019	
	*n* (*N* = 1468)	%	*n* (*N* = 1915)	%
Gender				
Female	1213	82.6%	1635	85.4%
Male	255	17.4%	275	14.4%
Gender diverse	0	0%	5	0.3%
Ethnicity				
Asian	29	2.0%	74	3.9%
Māori	251	17.1%	386	20.2%
New Zealand European	1017	69.3%	1192	62.2%
Pacific Islander	25	1.7%	55	2.9%
Other	146	9.9%	208	10.9%
New Zealand Index of Socioeconomic Deprivation score, mean	6.4		6.1	
Age, years, mean	37.6 (s.d. 12.8)		33.6 (s.d. 12.7)	

In 2014, individuals with a diagnosis of borderline personality disorder were dispensed between 0 and 15 psychotropic medications. Of those dispensed any medication, 49.9% were dispensed three or more psychotropic medications and 8.4% were dispensed seven or more psychiatric medications.

In 2019, the number of psychotropic medications dispensed to individuals with borderline personality disorder ranged from 0 to 18 medications. The percentage of individuals who were dispensed any medication who were on polypharmacy was 55.9%, and the percentage on seven or more medications was 10.7%. Both of these percentages represent statistically significant increases (*P* < 0.001 and *P* = 0.023, respectively) compared with the 2014 cohort.

**Fig. 1:**
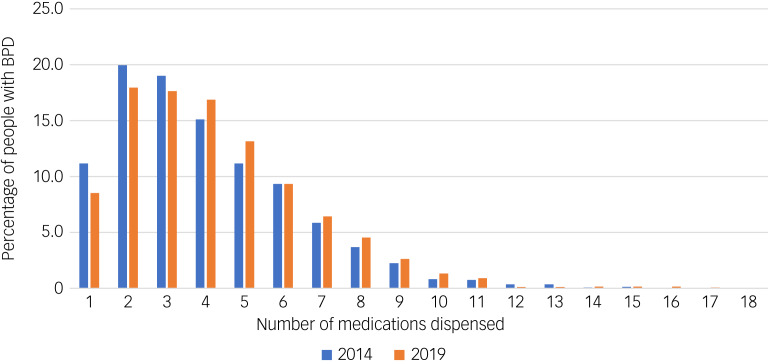
Number of psychotropic medications dispensed. BPD, borderline personality disorder.

Figure 1 shows the percentage of people with borderline personality disorder that were dispensed each number of psychotropic medications. The figure demonstrates a shift toward greater dispensing of psychotropic medications over time.

Medication dispensing increased between 2014 and 2019 for every medication class apart from treatments for substance use disorder. The most dispensed medication classes were antidepressants, followed by antipsychotic medications. Antidepressants were dispensed to 77.2% of those with a diagnosis of borderline personality disorder who were dispensed any medication in 2014, and 80.2% in 2019. Antipsychotics were dispensed to 63.8% in 2014 and 80.2% in 2019. Benzodiazepines and opioids were also commonly dispensed. Table 2 reports medication dispensing by class.

**Table 2:** Medicine dispensing by class (frequency and percentage of individuals)

	2014		2019	
	*n*	%	*n*	%
Antidepressants	1133	77.2%	1538	80.2%
Antipsychotics	936	63.8%	1288	67.3%
Benzodiazepines	812	55.3%	1123	58.6%
Mood stabilisers	422	28.7%	646	33.7%
Opioids/opiates	655	44.6%	882	46.1%
Non-benzodiazepine anxiolytics/hypnotics	16	1.1%	32	1.7%
Stimulants	39	2.7%	97	5.1%
Treatments for substance use disorder	271	18.5%	266	13.9%

Quetiapine was the most dispensed medication. It was dispensed to 49.4% of those with a borderline personality disorder diagnosis who were dispensed any medication in 2014, and 53.8% in 2019.

The largest change in dispensing was for lorazepam, which increased from 15.5% in 2014 to 26.7% in 2019 (*P* < 0.001). A full breakdown of individual psychotropic medications dispensed can be found in Appendix 2.

## Discussion

To our knowledge, this is the first study to report polypharmacy in a national cohort of people with borderline personality disorder. Psychotropic polypharmacy in people with borderline personality disorder was high, and increased between 2014 and 2019. Our findings were broadly consistent with cohorts of psychiatric out-patient clinics and hospitals in Europe and the USA.^3,4^ In a USA cohort of people with borderline personality disorder who required psychiatric hospital admission, 40% were on three or more medications.^10^ Two Spanish cohorts of out-patients reported that 47 and 50% were on three or more medications, respectively.^3,4^ In a British cohort of out-patients with an ICD-10 diagnosis of emotionally unstable personality disorder, two-thirds were on at least two psychotropic medications.^5^

Psychotropic polypharmacy could be partially explained by the high rates of psychiatric comorbidity, with 84.5% of people with borderline personality disorder meeting criteria for another psychiatric disorder in a community sample.^11^ The most common comorbidities were mood disorders, anxiety disorders and substance use disorders,^12^ which may explain the high rates of antidepressant dispensing in this group.

People with personality disorders may be less responsive to pharmacological treatment for comorbid depression, anxiety and post-traumatic stress disorder.^13,14^ Their reported symptom burden remains higher after treatment than those without personality disorders.^14^ When faced with continued patient distress and modest responses to treatment, it can be tempting for clinicians to add new medications despite limited evidence of efficacy. This may lead to psychotropic polypharmacy.^15^

The high burden of sedative medications, in particular quetiapine and lorazepam, were notable. This may be because these medications are utilised during periods of crisis as a means of managing acute distress or challenging behaviours.^16^ NICE guidelines acknowledge that this approach may be helpful in a crisis, but recommend prescribing a single sedative medication for no longer than a week.^1^

An increase in the dispensing of lorazepam from 15.5% in 2014 to 26.7% in 2019 is concerning. Rather than improving emotional regulation, benzodiazepines may worsen affective instability and impulsivity in this group.^17^ People with borderline personality disorder are more vulnerable to developing benzodiazepine misuse and dependence,^18^ and find it more difficult to reduce hazardous use.^19^

There was persistently high opioid dispensing, which increased between 2014 and 2019 from 44.6 to 46.1%. People with borderline personality disorder are more likely to develop chronic pain.^20^ However, opioids are no longer recommended for the treatment of non-malignant chronic pain,^21^ and people with borderline personality disorder have greater odds of developing a prescription opioid use disorder.^22^

Dispensing of antipsychotics and mood stabilisers remained high. Antipsychotic medications are associated with neurological side-effects such as extrapyramidal side-effects, acute dystonia and tardive dyskinesia.^23^ In addition, the metabolic effect of antipsychotic medication can lead to increased weight gain and increased risk of developing diabetes.^23^ Rarely, antipsychotics have potential fatal adverse effects such as myocarditis and agranulocytosis.^23^ Mood stabilisers commonly cause drowsiness and weight gain. Sodium valproate and carbamazepine are contraindicated in woman of childbearing age because of their teratogenicity.^23^

Psychedelics are not currently prescribed in New Zealand. There has been increased excitement about their potential in borderline personality disorder; however, at this time there is insufficient evidence to recommend their use in clinical practice.^24^

Polypharmacy may have negative psychological effects too. Widespread use and overreliance on psychotropic medications have been interpreted by some patients as reflecting clinicians’ lack of understanding regarding borderline personality disorder and a generally negative attitude toward patients with borderline personality disorder.^15^

A shift in treatment focus toward psychological interventions may be part of the solution.^25^ To achieve this, improved access to evidence-based psychological treatment for borderline personality disorder is required.^26^ In addition to the potential adverse effects of polypharmacy on the body, there is a significant financial burden associated with excessive prescribing for people with borderline personality disorder.^27^ Ideally, some of this resource would be redirected to evidence-based psychological treatment in the future.

The strength of this study was its use of national databases to achieve a broad overview of current clinical practice. This ensured that all patients with a diagnosis of borderline personality disorder by specialty mental health and addiction services were included. The Pharmaceutical Collection database captured all dispensing of prescription medications.

The study was limited by the quality of diagnostic reporting. The point prevalence of borderline personality disorder in community samples using structured interviews is approximately 1%.^28^ The diagnosis was captured at a much lower rate within New Zealand's national databases. A large portion of people with borderline personality disorder may be undiagnosed. Diagnoses made in primary care or by private psychiatrists would not have been captured unless those individuals had contact with publicly funded specialist services. This suggests that our study has captured the most severe disorders. The number of people diagnosed with borderline personality disorder increased from 1932 (2009–2014) to 2368 (2015–2019), but this does not necessarily represent an increase in the prevalence of the disorder. It may be attributable to increased recognition of the disorder among clinicians. We did not receive data on other primary or secondary diagnosis and are therefore unable to report the extent of comorbidity in this group. It is unknown what proportion of the polypharmacy reported in this study was indicated to treat other conditions.

In conclusion, this is the first study to use national data to look at polypharmacy in people with borderline personality disorder. More than half of individuals diagnosed with borderline personality disorder who were dispensed medication were on psychotropic polypharmacy (three or more psychotropic medications), and one in ten were on extensive polypharmacy (seven or more psychotropic medications) in 2019. Psychotropic polypharmacy increased between 2014 and 2019. This is concerning given the lack of evidence for pharmacological treatments in this group. Excessive prescribing has a negative psychological effect on patients^15^ in addition to the side-effect burden associated with polypharmacy.^24,29^ Our findings suggest that psychotropic polypharmacy is a significant burden for those with borderline personality disorder.

## Data Availability

Deidentified data from this study is available from the corresponding author, M.T., on request.

## Appendix 1 Psychotropic medications named in the national pharmaceutical dispensing data for New Zealand

**Table:**

Class	Medications
Antidepressants	Amitriptyline; clomipramine hydrochloride; dosulepin [dothiepin] hydrochloride; imipramine hydrochloride; maprotiline hydrochloride; nortriptyline hydrochloride; tranylcypromine sulphate; moclobemide; citalopram hydrobromide; escitalopram; fluoxetine hydrochloride; paroxetine; sertraline; mirtazapine; venlafaxine
Antipsychotics, general	Amisulpride; aripiprazole; chlorpromazine hydrochloride; clozapine; haloperidol; levomepromazine; levomepromazine hydrochloride; olanzapine; pericyazine; quetiapine; risperidone; ziprasidone; zuclopenthixol hydrochloride
Antipsychotics, depot injection	Flupenthixol decanoate; haloperidol decanoate; olanzapine; paliperidone; risperidone; zuclopenthixol decanoate
Mood stabilisers	Lithium carbonate; carbamazepine; gabapentin; lamotrigine; pregabalin; sodium valproate; topiramate
Benzodiazepines and zopiclone	Temazepam; triazolam; midazolam; nitrazepam; clonazepam; diazepam; lorazepam; oxazepam; zopiclone
Other anxiolytics and hypnotics	Melatonin; buspirone hydrochloride; phenobarbitone sodium;
Opioids and opiates	Codeine phosphate; dihydrocodeine tartrate; fentanyl; methadone hydrochloride; morphine hydrochloride; morphine sulphate; oxycodone hydrochloride; paracetamol with codeine; pethidine hydrochloride; tramadol hydrochloride
Stimulants	Atomoxetine; dexamfetamine sulfate; methylphenidate hydrochloride; methylphenidate hydrochloride extended-release; modafinil
Treatments for substance dependence	Buprenorphine with naloxone; bupropion hydrochloride; disulfiram; naltrexone hydrochloride; nicotine; varenicline tartrate

## Appendix 2 Individual medications dispensed to people diagnosed with borderline personality disorder who fulfilled a prescription in 2014 and 2019

**Table:**

	2014	2019
Buprenorphine with naloxone	0.5%	0.8%
Nitrazepam	0.5%	0.4%
Amisulpride	1.5%	1.1%
Amitriptyline	10.1%	7.8%
Aripiprazole	3.4%	6.6%
Atomoxetine	0.1%	0.4%
Bupropion	4.2%	4.6%
Buspirone	1.1%	1.5%
Carbamazepine	3.1%	1.7%
Chlorpromazine	2.1%	1.0%
Citalopram	17.8%	8.7%
Clomipramine	0.6%	0.5%
Clonazepam	13.6%	12.9%
Clozapine	1.3%	0.7%
Codeine	23.5%	26.9%
Dexamfetamine	0.1%	0.2%
Diazepam	12.3%	14.0%
Dihydrocodeine	2.4%	1.5%
Disulfiram	2.0%	1.9%
Dosulepin	0.3%	0.2%
Escitalopram	5.9%	11.3%
Fentanyl	0.7%	0.6%
Fluoxetine	11.2%	10.7%
Flupenthixol decanoate	0.4%	0.4%
Gabapentin	5.7%	8.0%
Haloperidol	1.8%	1.0%
Haloperidol decanoate	0.2%	0.1%
Imipramine	0.3%	0.1%
Lamotrigine	6.5%	10.6%
Levomepromazine	1.4%	1.6%
Lithium carbonate	4.6%	4.6%
Lorazepam	15.5%	26.7%
Melatonin	0.1%	0.2%
Methadone	2.0%	1.5%
Methylphenidate	2.5%	4.5%
Midazolam	0.5%	0.5%
Mirtazapine	8.6%	13.9%
Moclobemide	1.1%	0.7%
Modafinil	0.0%	0.1%
Morphine	5.7%	5.5%
Naltrexone	0.9%	1.0%
Nicotine	14.3%	9.9%
Nortriptyline	6.4%	6.2%
Olanzapine	10.8%	11.3%
Oxazepam	0.6%	0.5%
Oxycodone	3.1%	2.3%
Paliperidone	0.6%	1.5%
Paracetamol with codeine	11.4%	10.0%
Paroxetine	5.7%	3.5%
Pericyazine	0.3%	0.3%
Pethidine	0.1%	0.1%
Pregabalin	0.0%	5.3%
Quetiapine	49.4%	53.8%
Risperidone	7.3%	7.9%
Sertraline	7.6%	19.9%
Sodium valproate	11.7%	8.1%
Temazepam	7.4%	3.3%
Topiramate	2.3%	3.0%
Tramadol	19.2%	19.7%
Tranylcypromine	0.3%	0.1%
Triazolam	1.4%	1.1%
Varenicline	3.1%	1.5%
Venlafaxine	25.3%	22.9%
Ziprasidone	0.8%	0.6%
Zopiclone	33.5%	33.9%
Zuclopenthixol	0.2%	0.6%
Zuclopenthixol decanoate	0.7%	0.5%
